# Guidelines for genetic ancestry inference created through roundtable discussions

**DOI:** 10.1016/j.xhgg.2023.100178

**Published:** 2023-01-13

**Authors:** Jennifer K. Wagner, Joon-Ho Yu, Duana Fullwiley, CeCe Moore, James F. Wilson, Michael J. Bamshad, Charmaine D. Royal, Genetic Ancestry Inference Roundtable Participants

**Affiliations:** 1 School of Engineering Design and Innovation, Pennsylvania State University, University Park, PA 16802, USA; 2 Institute for Computational and Data Science, Pennsylvania State University, University Park, PA 16802, USA; 3 Department of Biomedical Engineering, Pennsylvania State University, University Park, PA 16802, USA; 4 Rock Ethics Institute, Pennsylvania State University, University Park, PA 16802, USA; 5 Penn State Law, University Park, PA 16802, USA; 6 Huck Institutes of the Life Sciences, Pennsylvania State University, University Park, PA 16802, USA; 7 Department of Pediatrics and Institute for Public Health Genetics, University of Washington, Seattle, WA 98195, USA; 8 Treuman Katz Center for Pediatric Bioethics, Seattle Children’s Hospital and Research Institute, Seattle, WA 98101, USA; 9 Department of Anthropology, Stanford University, Stanford, CA 94305, USA; 10 The DNA Detectives, Dana Point, CA, USA; 11 Centre for Global Health Research, Usher Institute, University of Edinburgh, Edinburgh EH8 9AG, Scotland; 12 Department of Pediatrics and Department of Genome Sciences, University of Washington, Seattle, WA 98195, USA; 13 Division of Genetic Medicine, Seattle Children’s Hospital, Seattle, WA 98101, USA; 14 Departments of African and African American Studies, Biology, Global Health, and Family Medicine and Community Health, Duke University, Durham, NC 27708, USA

**Keywords:** genetic ancestry, population descriptors, race and ethnicity, ELSI

## Abstract

The use of genetic and genomic technology to infer ancestry is commonplace in a variety of contexts, particularly in biomedical research and for direct-to-consumer genetic testing. In 2013 and 2015, two roundtables engaged a diverse group of stakeholders toward the development of guidelines for inferring genetic ancestry in academia and industry. This report shares the stakeholder groups’ work and provides an analysis of, commentary on, and views from the groundbreaking and sustained dialogue. We describe the engagement processes and the stakeholder groups’ resulting statements and proposed guidelines. The guidelines focus on five key areas: application of genetic ancestry inference, assumptions and confidence/laboratory and statistical methods, terminology and population identifiers, impact on individuals and groups, and communication or translation of genetic ancestry inferences. We delineate the terms and limitations of the guidelines and discuss their critical role in advancing the development and implementation of best practices for inferring genetic ancestry and reporting the results. These efforts should inform both governmental regulation and self-regulation.

## Teaser

Two roundtables engaging a diverse group of stakeholders enabled the development of guidelines for inferring genetic ancestry in academia and industry. This report describes the engagement process and the resulting guidelines, which can inform continued improvement of practices and assist ongoing scholarly, industry, and policy discussions.

## Introduction

Genetic ancestry inference is commonly used by researchers (e.g., to control for population stratification, explore population history, etc.), sought by individuals via direct-to-consumer (DTC) genetic testing companies to learn more about their genealogical history, geographic origins, and population affiliations, and contemplated by precision health initiatives as a means to “return value” or otherwise engage patient-participants. Both research findings and individual DTC results are often featured in the print and broadcast media. Genetic ancestry inference is thus used across a wide variety of settings, with different goals, assumptions, methods, and claims of accuracy and validity. It is therefore not surprising that there has been considerable disagreement among diverse stakeholders about the merits, limitations, utility, and impact of genetic ancestry inference. Consequently, efforts by any individual stakeholder group to advocate for standards or regulation have met with little success. The need for standards and other guidance has never been greater, as the popularity of DTC genetic ancestry testing continues to expand,^1^ members of Congress have expressed concern,^2^ controversial applications of genetic ancestry information continue to be explored (e.g., forensic or investigative genetic genealogy;^3^^,^^4^^,^^5^^,^^6^^,^^7^^,^^8^ immigration decisions;^9^^,^^10^ political posturing;^11^^,^^12^ medical school admissions;^13^ entitlement eligibility;^14^^,^^15^ and social identity and belonging^16^^,^^17^^,^^18^^,^^19^), and society has little choice but to confront whether, how, and when it is appropriate to rely upon anyone’s genetic ancestry information.

In 2008 the American Society of Human Genetics (ASHG) convened a task force to study issues emerging from genetic ancestry inference in academia and industry and to explore a constructive response to the need for more transparency and discussion about what genetic ancestry inference entails, as well as how it works and what it can and cannot do. The ASHG Board of Directors approved an ASHG position statement developed by the task force and the Society’s Social Issues Committee on ancestry inference that called attention to motivations, technical accuracy and reliability of results, health implications, societal and personal implications, proprietary databases, communication of limitations and potential impacts, public and personal education, interdisciplinary collaboration, and mechanisms for accountability (https://www.ashg.org/wp-content/uploads/2008/11/Statement-20081311-ASHGAncestryTesting.pdf) and approved an ASHG White Paper that provided recommendations aimed at addressing these issues.^20^

One of the recommendations of the task force was to convene a diverse group of stakeholders and develop guidelines for genetic ancestry inference that would inform research and commercial applications of ancestry testing. The long-term goal was to develop the precursors to best practices for inferring genetic ancestry and guidelines for the uses and reporting of genetic ancestry testing results. A planning committee was formed consisting of two Chairpersons (C.D.R. and M.J.B.), representatives of human population geneticists, the DTC ancestry testing industry, scholars in ethical and social implications of human genetics, and consumers of ancestry testing (e.g., genetic genealogists), and it organized a “roundtable” to develop consensus statements and guidelines for genetic ancestry inference. A first meeting (i.e., Roundtable I) was held in Washington, DC in September 2013. There were 65 participants representing seven stakeholder groups: population geneticists, DTC ancestry testing companies, social and behavioral scientists, humanists, clinicians, community representatives, and consumer groups including genealogical associations/societies. The participants generated 22 “consensus” statements—defined as statements accepted by a majority but not all participants—about the uses, methods, and outcomes of genetic ancestry inference (Table 1). Critical dissent and constructive criticisms were encouraged to ensure that all stakeholders had a “voice” and that multiple perspectives—even those that were highly dissenting—were considered. Reports from those in the minority were solicited. Several statements were edited for clarity and grammar in subsequent email exchanges among participants. A second roundtable meeting (i.e., Roundtable II) was held in Durham, NC in May 2015 to compose, based upon these 22 consensus statements, a set of guidelines (Table 2) for ancestry inference that would serve as a template for the development of best practices. There were 49 participants in this second meeting, including 40 attendees from the first roundtable meeting.

**Table 1:** Stakeholder group views: Consensus statements and voting summary

Consensus statements	Voting results (% yes/% no; no. of votes)
1. Genetic ancestry inference uses regions of the genome that inform us about individual, genealogical, population, and/or geographical ancestry, by comparison of similarities to a reference sample(s). There are many different types of ancestry tests that use Y DNA, mtDNA, autosomal, and/or X chromosome markers. Information about parentage, kinship, and identification can be captured by ancestry tests and vice versa. This information can also be used for forensic applications.	90/10; n = 48
2. Whether an application of genetic ancestry testing is appropriate or not is context-dependent.	90/10; n = 48
3. Individuals may pursue genetic ancestry testing and should be informed of the potential impact of their results on themselves, their family members, and their communities.	85/15; n = 48
4. Genetic ancestry may inform our understanding of disease risk and guide treatment options in research and clinical settings.	90/10; n = 48
5. It is recommended that genetic ancestry tests alone not be used to associate people with culturally, legally, or socially defined groups.	78/22; n = 51
6. Genetic ancestry results alone are not a valid basis for denying individuals’ or peoples’ human rights, responsibilities, or social benefits.	73/27; n = 48
7. Ancestry testing should be conducted with standards (to be defined) and the data/methods/models used should be transparent, ideally publicly available, to promote accuracy and facilitate interpretation.	94/6; n = 53
8. A reference sample is composed of individuals sampled from contemporary populations that are assumed to share recent common ancestry with an ancestral group or lineage.	87/13; n = 53
9. How representative a contemporary population is of an ancestral group depends in part on the age and history of the ancestral group.	82/18; n = 49
10. There are multiple sources of genetic information about ancestral populations including existing populations, ancient remains, and statistically inferred group.	96/4; n = 51
11. Development of minimum standards and best practices for genetic ancestry inference is needed.	92/8; n = 51
12. Researchers and providers of ancestry tests should identify limitations of their genetic ancestry inference and make these clear to the general public, research participants, and consumers.	89/11; n = 53
13. Genetic ancestry inference is a tool for individuals to ask personally meaningful questions and discover new information about themselves, their family, and their ancestors.	96/4; n = 48
14. Many benefits and harms of genetic ancestry inference are dependent on personal experiences and perceptions of identity, society’s perception of an individual, and community context.	92/8; n = 50
15. There are multiple perceived individual benefits to genetic ancestry inference including satisfying curiosity, assisting in identification, inclusion as a member of a group, discovery of kinship and ancestry information, potentially conferring status, connection to historical and evolutionary context, and facilitating investigation of health risks and treatment options.	96/4; n = 47
16. For communities, including researchers, benefits include understanding heritage and group history, as well as facilitating investigation of population disease burden. More broadly, engagement with ancestry testing can potentially promote a broader understanding of genetics and human evolution.	96/4; n = 49
17. Genetic ancestry testing enhances our understanding of human history and advances scientific research by the large volume of data being generated and crowd-sourcing of analysis.	90/10; n = 49
18. Genetic ancestry testing generates collaborative relationships and cross-fertilizes ideas between researchers, industry and non-scientists; creates more training and employment opportunities for individuals; engages under-represented communities in science; benefits development of academic and industrial enterprises; and promotes science and history education, citizen science, and bioethical innovations.	84/16; n = 50
19. Genetic ancestry testing provides information and knowledge. Interpretation and application of data from genetic ancestry tests can, depending on context, lead to benefit or harm.	86/14; n = 50
20. The frequency and magnitude of both benefits and harms of genetic ancestry testing are largely unknown and require systematic investigation.	82/18; n = 49
21. Potential group harms of genetic ancestry testing include stigmatization; challenging origin narratives and/or religious beliefs; erroneous or inconsistent results undermining the credibility of science and medicine; reinforcing biologically deterministic notions of race and ethnicity; and a false sense that one population is more evolved than another.	78/22; n = 49
22. Ancestry testing has the potential to personalize (e.g., emotional distress) the violence of historical atrocities (e.g., slavery in the U.S.).	78/22; n = 50

**Table 2:** Summary of voting results for guidelines

Guideline	Vote	Confidence level (extremely, very, somewhat, not at all)			
1. Application of genetic ancestry inference	for: 26 (76%)	6	11	9	0
	against: 5 (15%)	2	1	1	1
	abstain: 3 (9%)				
2. Assumptions and confidence/laboratory and statistical methods	for: 28 (82%)	9	15	4	0
	against: 3 (9%)	0	2	0	1
	abstain: 3 (9%)				
3. Terminology and population identifiers	for: 28^a^ (82%)	13	11	3	0
	against: 4 (12%)	1	1	1	1
	abstain: 2 (6%)				
4. Impact on individuals and groups	for: 29^a^ (85%)	9	13	6	0
	against: 3 (9%)	1	0	1	1
	abstain: 2 (6%)				
5. Communication/translation of genetic ancestry inferences	for: 29 (85%)	12	7	10	0
	against: 2 (6%)	0	1	0	1
	abstain: 3 (9%)				

a One “for” response did not indicate level of confidence for guidelines 3 and 4.

Here we report on these efforts before discussing emergent issues and offering suggestions for next steps and insights that could inform both governmental regulation and self-regulation.

## “Consensus” statements (Roundtable I)

The planning committee produced a set of eight draft consensus statements and related questions focused on eight key areas: (1) the application of genetic ancestry inference; (2) assumptions underlying genetic ancestry inference; (3) laboratory and statistical methods; (4) estimating confidence and communicating uncertainty; (5) terminology and population identifiers; (6) assessing and addressing individual and group benefits; (7) assessing and addressing individual and group harms; and (8) translating genetic ancestry findings for the media and public. These statements were distributed to attendees of Roundtable I prior to the meeting and served as starting points for discussion. Roundtable I was structured into eight sessions of small group discussions followed by negotiation and adjudication, and each attendee was allotted one vote in a general session of all attendees. Each small group consisted of representatives from each stakeholder category. Their discussions revolved around questions provided to them about each draft consensus statement. They were instructed to accept the statement as is, edit the statement, or develop a new consensus statement.

Roundtable I resulted in an expansion of the eight draft consensus statements to 22 (Table 1), reflecting robust discussion and reactions to the questions that accompanied the draft statements. Clearly, it was often the case that discussion identified that original draft statements did not adequately capture the complexity of the questions posed and therefore required further elaboration. In several instances a consensus statement was deemed obvious, and therefore unnecessary, to one stakeholder group but was simultaneously judged by another stakeholder group to be unobvious and important. Eighty-five percent or more of attendees agreed with 15 statements, and no statement received less than 73% of positive votes (Table 1). Dissent from the consensus statements was captured by minority reports that were solicited from attendees at the conclusion of Roundtable I. In general, these reports reflected continued ambivalence about wording, technical accuracy, and necessity of a consensus statement.

A reflective critique of Roundtable I revealed to the planning committee that the design might have unintentionally constrained the meeting’s potential for achieving its goal: the development of guidelines through consensus. Had the initial small group discussions been organized as an opportunity to first reach consensus within stakeholder groups and later work toward consensus between and among stakeholder groups in the full discussion, the process might have more readily identified the most serious gaps in understanding of the issues and underlying bases for divergent opinions. Instead, some participants made compromises to their positions within their small group discussion only to see those points rehashed in the full group discussion. Thus, this process was not as efficient, or satisfying to participants, as it otherwise might have been, and there is a possibility that the votes tallied on the consensus statements at the end of a protracted discussion reflected an indifference or disengagement of some participants rather than genuine support for any particular statement. Accordingly, the approach to Roundtable II was changed to improve the process and maximize the utility of the progress that was made in Roundtable I.

## Guidelines (Roundtable II)

Based on the consensus statements, minority reports, and feedback from and self-critique of Roundtable I, the planning committee produced a set of draft guidelines in six areas: (1) application of genetic ancestry inference; (2) assumptions and confidence underlying genetic ancestry inference; (3) laboratory and statistical methods; (4) terminology and population identifiers; (5) assessing and addressing impact on individuals and groups; and (6) communication/translation of genetic ancestry inferences. These draft guidelines were distributed to attendees of Roundtable II prior to the meeting and served as starting points for the discussion.

Roundtable II was structured differently from Roundtable I to facilitate conversation both within and between the stakeholder groups (social scientists/humanists/community representatives, genetic genealogists, ancestry testing companies, and geneticists). Specifically, in Roundtable II there were four sessions of stakeholder group discussions followed by four sessions of mixed-stakeholder groups and then a review in a plenary session with all attendees. As with Roundtable I, groups were instructed to accept each guideline as is, edit the guideline, or develop a new guideline. Roundtable II resulted in reduction of the six draft guidelines to five. More than 80% of attendees agreed with four guidelines, and no guideline received less than 76% of positive votes (Table 2). To capture dissent from the guidelines, minority reports were solicited from attendees at the conclusion of Roundtable II. No minority reports were received. Below we present each guideline accompanied by a summary of comments concerning the importance of the guideline, our reasons for including the guideline, further explanations that are needed, limitations of the guideline, and suggestions for best practices relevant to the guideline.

### 1. Application of genetic ancestry inference

#### GUIDELINE

Genetic ancestry testing can be used in myriad ways, for example, to estimate individual ancestry proportions, infer genealogical relationships among individuals, characterize population history, and adjust for population stratification in biomedical research. Researchers, study participants, ancestry testing companies, and consumers should be aware that many aspects of ancestry inference continue to change because of changing reference populations, type and number of marker sets, methods of analysis, and understandings of human history. As the process of ancestry inference evolves, interpretations may change. Genetic ancestry information is only one line of evidence for establishing or denying an individual’s or group’s eligibility for particular entitlements (e.g., affirmative action in education, citizenship, employment, etc.). Where they exist, other lines of evidence should also be considered.

This guideline is important for several reasons. While genetic ancestry inference is a moving target, consumers often think that “a test is a test” and that it offers definitive, static answers. This guideline recognizes the role that genetic ancestry inference does or might play in how individuals think about their racial or ethnic background and in informing individuals’ quests for entitlements as well as underscoring the many variables that contribute to why ancestry testing is capable of offering only partial information due, for example, to the limited type and number of markers tested, as well as the populations and time frame sampled. Genetic ancestry is not synonymous with genealogical ancestry, as an individual does not inherit genetic material from all of one’s distant ancestors, and the inheritance of genetic material from a particular distant ancestor need not be a prerequisite to, or preclude, a social affinity with that distant ancestor’s culture. The guideline explains why the nature of genetic ancestry information might be subject to redefinition, reclassification, or other changes. In this way, this guideline aims to prevent overstating results and, in the case of DTC companies, overselling information. The guideline further helps a broad readership understand the distinction between acting on information that is an “inference” versus treating the information as a “fact.” Genetic ancestry information (or “results”) is subject to change, but many people might not, for a variety of reasons, understand how, why, or what that means. Rather than indicating a problem, changes in an individual’s “results” over time, and even differences across DTC services, could reflect uncertainty in science, which at its best is a self-correcting way of knowing the world. For example, changes in an individual’s results could be attributed to analytical methods having improved or discoveries having occurred after the information was initially generated and/or communicated to the consumer or user. That the “result” is not a definitive or permanent answer is important when thinking about the appropriateness of decision-making based upon that information. Such decisions include, for example, setting a threshold proportional ancestry that must be met for group membership or acquiring citizenship based on “results” suggesting affiliation with one country that later does or could turn out to be less likely than affiliation with another country.

This guideline is included to provide context to the changing nature of the field to consumers and researchers alike; to acknowledge the potential benefits and harms to individuals and groups; to emphasize the wide-ranging applications of genetic ancestry testing; and to stress that caution should be taken when any entity communicates results. Genetic ancestry inference affects the public’s confidence in genetic testing in general and vice versa, even if the methods, analysis, and resulting information gleaned for the analysis and application are quite different. As noted by Nelson (2016, at 81), “DNA *spillover* occurs when an individual’s experience with one domain of genetic analysis informs his or her understanding of other forms of it or authorizes its use in another domain.”^21^ So, it is important to make assumptions underlying genetic ancestry inference transparent and understandable upfront and to distinguish the features of this type of genetic test so that genetic ancestry inference does not unjustifiably undermine or, alternatively, unnecessarily bolster the public’s confidence in or willingness to consider or trust other types of genetic information (e.g., results of genetic testing for Mendelian conditions).

Further explanation is needed for this guideline to clarify why test interpretations continue to change, and should be expected to continue to change, to provide examples of when ancestry inference has been used for determining eligibility for entitlements, and to elucidate points of tension across stakeholder groups regarding whether and when genetic ancestry information is ever appropriate without corroborating non-genetic evidence.

There are limitations to this guideline. Some might view it as a prelude to guidelines rather than a guideline itself. Additionally, it might not be clear or obvious to consumers how changes in testing or reference samples would impact interpretation. Current interpretations—particularly interpretations indicating admixture proportions—are not static and will change in the future in foreseeable and unforeseeable ways. Perhaps the most glaring limitation of this guideline is that it calls out four groups (i.e., researchers, study participants, ancestry testing companies, and consumers) and, in so doing, apparently overlooks how users (who can be, but are not necessarily, the purchasers or test-takers), bystanders, and others actively make decisions based on the genetic ancestry information, are passively influenced by the information, or unknowingly are affected—directly and indirectly—by what the four identified groups know, believe, and do.

This guideline lends itself to a few practical next steps for those engaged in genetic ancestry inference (which were not themselves addressed as part of the roundtable discussions). First, it is important to monitor DTC testing websites for updated information and compile that information for easy reference. While a particular actor—whether a specific individual or entity—to perform this task is not inherently obvious, an independent, impartial, and trustworthy actor (such as the International Society of Genetic Genealogy, ISOGG) would be appropriate. Second, we encourage the creation and use of a model disclosure form throughout the DTC genetic ancestry industry (similar to other consumer protection disclosure forms, such as “your credit score and understanding your credit score”) that includes the material information necessary to understand the genetic ancestry testing. Third, it is preferred that the research and commercial providers make educational materials available and discuss this information prior to or as an integral part of the informed consent and purchasing processes.

### 2. Assumptions and confidence/laboratory and statistical methods

#### GUIDELINE

The assumptions, type and number of genetic markers, and reference populations used for ancestry inference should be explicitly described. An easily interpretable assessment of confidence, accuracy, and limitations should accompany quantitative claims about genetic ancestry inference. Steps should be taken to adequately convey the amount and sources of uncertainty in the inferences about genetic ancestry, whether in a research or commercial setting. As a means of proficiency testing, providers of genetic ancestry inference should analyze samples against a standard reference population panel (e.g., 1000 Genomes) that represents human global diversity to the extent possible and should communicate the results. Scientifically acceptable standards should be established for validation of results. Individuals should be informed of the availability of their raw data or lack thereof.

The goal of this guideline is to offer transparency about how results are determined and to establish standards so that results can be replicated or validated. For example, there are many different ways to look at data as rich as human genetic variation, and a model or approach appropriate for one purpose and context could be misguided for another purpose or context depending upon the assumptions and constraints of that particular model or approach. This guideline is important to enable researchers as well as consumers to make informed decisions about genetic ancestry tests and to enable consumers and other users to interpret the test results within the proper context of the test’s limitations. Methods are not uniform from one researcher or DTC provider to another or from one type of test to another. Without disclosure of these material details, it is difficult for a person (whether study participant or consumer) to obtain a second opinion (that is truly an apples-to-apples comparison) or make sense of the information. This guideline, by encouraging the disclosure of this information, will aid in the identification of the “snake oil salesmen” among those offering genetic ancestry analyses to the public. This guideline is included to avoid misinterpretation and overinterpretation of genetic ancestry test or inference results.

Further explanation is needed for this guidance, as it does not define validation or what specific measures are expected or steps to be taken. First, the guideline does not articulate how proficiency testing would best or most reasonably be achieved. Establishing an accepted proficiency testing approach would require substantial work (Box 1). Additionally, the guideline does not stipulate what information about reference populations is necessary for meaningful disclosure. At the very least, transparency about reference populations used should include their size, geographic location, level of population inclusivity, and how the markers used in genetic ancestry panels were selected, validated, and sometimes excluded to optimize a workable model of homogeneity in the reference populations being compared.

Box 1 **Preliminary factors to consider when designing “proficiency testing” for genetic ancestry inferences**

• How is validation being defined and, relatedly, what specific measures are expected?
  ○ How do genetic ancestry inferences for each test company compare to one another?
  ○ How do genetic ancestry inferences for each test company compare to a “gold standard” (e.g., published literature) or benchmark (e.g., a set of agreed upon DNA samples, such as a subset of 1000 Genomes Project, or an agreed upon simulated dataset)?
• Does the proficiency testing process require companies to take a proficiency test from DNA to interpretation, or can the companies interpret existing genotypes?
  ○ If the former, the numbers need to be limited to avoid overburdening small companies (which would disproportionately feel the associated costs).
  ○ For proficiency testing to be effective, the process needs to have support and participation across the industry (from DNA ancestry testing companies with large and small market shares alike).
  ○ It seems feasible for those engaged in genetic ancestry inference to select a subset of 1000 Genomes Project samples that include diverse representation of samples with substantial admixture and samples from different geographic areas.
  ○ Companies would need to be blinded to the DNA IDs of the benchmark sample set to avoid foreseeable gaming of the proficiency testing system (e.g., by just looking up the 1000 Genomes Project publications).

Moreover, this guideline needs further explanation as to what is meant by “should communicate the results” and what is intended by “an easily interpretable assessment.” Without stating the perspective by which this should be judged (e.g., applying a “reasonable person standard” such as a hypothetical non-expert consumer), there might be widely varying ways of complying. One could envision an assessment being easily interpretable from one geneticist to another as satisfying the underlying purpose of this guideline; however, without more explanation, some might interpret this guideline as requiring a type of communication that goes beyond typical consumer protection requirements for formatting and text to address complex matters of both data literacy and scientific literacy in addition to basic literacy.

Limitations of this guideline are (1) that thresholds for acceptability, validity, and the like are not stated or given but would be needed for interpretation by consumers and (2) that there are many different types and sources of uncertainty, which are difficult to quantify. This guideline lends itself to at least two genetic ancestry inference practice suggestions. Establishing and sustaining a centralized resource where regular updates of these measures could be compiled and displayed in one trusted location for comparisons by consumers, study participants, and others would be meritorious. Additionally, interoperability practices should be developed and encouraged. Establishing a standardized file format for returning data to participants would enable another DTC provider or researcher to examine genetic ancestry inferences using different methods and offer “second opinions.”

### 3. Terminology and population identifiers

#### GUIDELINE

Those inferring genetic ancestry should clearly explain how they are using the terms pertaining to race, ethnicity, nationality, and other population identifiers. Those inferring ancestry should provide clear information on what reference samples are used to represent populations and what criteria are used for inclusion in a reference sample (e.g., self-identified ancestry, all grandparents from that population, etc.). Reference populations used to draw ancestral inferences should be labeled using specific geographic, cultural, and temporal identifiers.

This guideline is important because genetic ancestry inference has the simultaneous potential (1) to disconnect, alienate, and estrange individuals from groups and identities with which they are affiliated based on other types of evidence as well as (2) to connect and reconnect individuals, families, and communities with groups and identities to which they have always felt themselves to belong, or, on the other hand, to provide a bridge to peoples with whom they have no current affiliation. These disconnections, new connections, and reconnections could have real consequences in terms of how individuals and institutions understand cultural identities and define social rights and obligations as well as political responsibilities. This guideline underscores that inaccurate or even sometimes colloquial association of various terms with “ancestry” has the potential to lead consumers and the broader public to incorrect conclusions about socially ascribed populations, especially traditionally marginalized populations. In the absence of consistent terminology, and an acknowledgment that populations have complex histories of mixing and migration that should also be noted alongside their present-day appellations, consumers are sometimes confused about differences in tests that produce varying results. Increasing popularity of DTC genetic testing offers an opportunity to engage and educate the public, and common terminology and identifiers would be useful. This guideline emphasizes that accurate population labeling should include geographic, cultural, and temporal identifiers to acknowledge that these aspects of human identity are also important and that they are not static or fixed but, rather, have changed and will continue to change over time.

Identifiers are not uniform across geopolitical boundaries and do not remain constant over time. The instability of identifiers in the United States Census is well known, and common practices to aggregate and disaggregate distinct concepts of ethnicity, country of origin, and nationality into one or more Office of Management and Budget (OMB) race categories or to synthesize global data further complicate the matter (e.g., OMB,^22^^,^^23^ Institute of Medicine,^22^^,^^23^^,^^24^^,^^25^^,^^26^^,^^27^^,^^28^ Morning,^22^^,^^23^^,^^24^^,^^25^^,^^26^^,^^27^^,^^28^ Villarroel et al.,^22^^,^^23^^,^^24^^,^^25^^,^^26^^,^^27^^,^^28^ Popejoy et al.,^22^^,^^23^^,^^24^^,^^25^^,^^26^^,^^27^^,^^28^ Byeon et al.^22^^,^^23^^,^^24^^,^^25^^,^^26^^,^^27^^,^^28^). Terminology used with genetic ancestry inference must be as clear, accurate, and precise as possible to promote understanding. For example, rather than describing a DNA sample or data with merely a regional geographic descriptor (e.g., West African), it might be useful to have additional information such as self-identification (e.g., Yoruba), place of collection (e.g., Ibadan, Nigeria), and language or dialect. It would also be helpful if, to the extent practicable, samples and reference populations were accompanied by a researched description providing further reading for users on the historical emergence of each reference population. This step would help to preclude tendencies that “fossilize” groups as static, which is especially needed to broaden conceptions of non-Western and marginalized peoples. Such descriptions might include dynamics that have shaped groups’ complex histories and current social practices. This is to emphasize Ogundiran’s point regarding the history of the Yoruba: that identity making is a process (see, e.g., Ogundiran,^29^^,^^30^^,^^31^ Law,^29^^,^^30^^,^^31^ and Matory^29^^,^^30^^,^^31^). This guideline is included to ensure that consumers can make informed choices about different genetic ancestry tests and better understand why test results might differ between platforms. Moreover, its inclusion reflects the roundtable participants’ recognition that misinterpretation and overgeneralizations, enabled by loose and inconsistent terminology unmoored from history, can contribute to misunderstandings of other people, social discrimination, and racism that must be acknowledged, prevented, and mitigated.

Further explanation of the underlying rationale for this guideline is needed to articulate why social labels can be unintentionally confusing and their use potentially harmful in the context of genetic ancestry inference. For example, DTC genetic ancestry inference reporting a presence or absence of African ancestry or Native American ancestry can disrupt one’s self-identity. Additionally, further explanation is needed as to what specific geographic, cultural, or temporal identifiers are appropriate, how often those labels should be revisited, and how their appropriateness is to be assessed.

While this guideline has the notable limitation that enforcement of such practices and disclosures would be challenging, the genetics community’s support of this practice could create sufficient normative pressure to encourage its implementation. Additionally, this guideline could encourage best practices if an authoritative, trustworthy entity were to maintain and host a compilation of nomenclature regarding this information for available DTC genetic ancestry tests and prominent research efforts.

Subsequent to the roundtable discussions, the National Academies of Sciences, Engineering, and Medicine (NASEM) took steps toward developing and recommending best practices for use of population descriptors not only in genetic ancestry inferences but also in human genetics and genomics research more broadly. In 2021, NASEM established an ad hoc committee tasked with examining population descriptors (such as race, ethnicity, and genetic ancestry) in genomics research and offering “best practices” including recommendations for “culturally responsive methods and common data elements” that could enable the harmonization of population descriptors used in genomics research within and beyond the United States.^32^

### 4. Impact on individuals and groups

#### GUIDELINE

Individuals pursuing ancestry inference should be informed of potential impacts on themselves, family members, and communities. They should also be informed of the potential for planned and unplanned access to their genetic ancestry information by law enforcement, government agencies, commercial entities, and other third parties. Genetic ancestry inference is an international activity; thus, people should be aware of the different regulations regarding privacy and data security across countries. Genetic ancestry inference can lead to the discovery of unexpected results that conflict with existing beliefs or knowledge. Potential positive and negative impacts should be laid out prior to testing or participation in research. Individuals should consider discussions with family members prior to undertaking genetic ancestry inference because it may have implications for relatives. Those inferring genetic ancestry should facilitate the study of potential individual- or group-level impacts to inform the development and implementation of best practices for communicating potential impacts of ancestry inference. The potential outcomes should be broadly disseminated.

This guideline’s importance is to protect consumers and study participants from the complex web of third-party interests and varying regulations that intersect with genetic ancestry testing and information. It is intended to enable consumers to make informed decisions about genetic ancestry tests and sharing their data directly with others, whether within an ancestry testing company’s website or more broadly through online publication elsewhere. The guideline also is important in its recognition that there are potential benefits and risks of harm that can occur at the individual level and at familial, community, or broader social levels. For example, for groups in the United States that have been oppressed and had their “roots,” cultures, and actual ancestors purposefully eradicated, actively erased, or historically concealed from them, the acquisition of genetic ancestry information could affect not only the connections that individuals make with the past but also the viability of connections that individuals pursue in the present and future. Unexpected outcomes from DTC genetic testing are not infrequent, and, in most cases, these outcomes involve kinship rather than ancestral origins (e.g., Law^30^). Additionally, this guideline is important because ancestry inference results might be unexpected or disruptive to an individual’s or group’s understanding of themselves. Individuals and families should be aware of the limitations to genetic privacy and understand that their DNA samples or data might be used in other contexts, such as law enforcement or other governmental agencies (e.g., Guerrini et al.^3^). (While law enforcement agencies’ use of resources made available by DTC genetic ancestry testing was a possibility at the time the roundtables were held, it was not until years after the roundtables that news of investigative genetic genealogy to catch California’s Golden State Killer brought widespread media attention to the issue [e.g., Zhang,^33^^,^^34^ Speakers et al.^33^^,^^34^)

This guideline reflects the roundtable participants’ recognition (1) of variation in perceived and potential benefits and risks associated with genetic ancestry testing that might impact either individuals or socially-ascribed groups and (2) that it is an ethical responsibility to communicate these possibilities to the potential test-takers prior to the DNA collection and analysis. The issue of third-party uses of ancestry data and the variation in regulations governing data security and privacy increase the potential risk of harm to consumers and the need for protections. Related to this is the pervasive commercial interest in the digital age that requires consumer confidence in mechanisms for privacy and consensual use of data perceived as personal information. Following the roundtables of 2013 and 2015, considerable privacy law scholarship and data protection policy developments have materialized in the genetics community^35^^,^^36^^,^^37^^,^^38^^,^^39^^,^^40^^,^^41^^,^^42^^,^^43^^,^^44^^,^^45^^,^^46^^,^^47^^,^^48^^,^^49^^,^^50^ and society more broadly. These developments, discussed further below, have ongoing implications for the responsible collection, storage, and use of genetic and genomic data.

Notably, this guideline is an aggregate of several related but distinct components that the roundtable participants conceptualized under the rubric of potential risks and benefits. The broad scope or apparent randomness of this guideline’s aggregated components might limit its effectiveness. A reordering of the topics or offering of subcategories might be helpful to clarify the meaning. Additionally, the impacts (real and perceived; possible and actualized) are emerging continuously and might not be immediately apparent, obvious, or noticed by those inferring genetic ancestry or seeking genetic ancestry inferences. Another potential limitation of this guideline is that privacy and third-party data use issues are broader than the ancestry-specific context, so there is potential for these recommendations to be subsumed within existing best practices regarding genetic information more generally.

There are several potential next steps prompted by this guideline. Firstly, the research community, the DTC ancestry testing companies, and/or another entity should compile and provide analyses of applicable laws and regulations regarding access and use of genetic ancestry information from the state and federal levels in the United States and international jurisdictions. Secondly, the potential benefits and risks for groups of people should be presented to prospective study participants and consumers in addition to potential benefits and risks for the individual. Thirdly, the final two sentences of this guideline impose a directive that geneticists and social scientists work collaboratively and, moreover, suggest that when genetic ancestry inference is being conducted as research it would be good practice to integrate pre- and post-assessment of outcomes and enable ongoing communications with participants (in other words, a longitudinal and more engaged approach is preferred). Another good practice is to identify conspicuously (i.e., clearly and prominently) the major issues for those having their genetic ancestry inferred and also to promote an informed consent or informed purchase process that prompts the individual (whether study participant or consumer) (1) to review the aforementioned personal, familial, social, data, and oversight considerations and (2) to communicate with family members about their intention prior to obtaining the genetic ancestry information.

### 5. Communication/translation of genetic ancestry inferences

#### GUIDELINE

Genetic ancestry inference should be communicated in ways that are as accurate and understandable as possible for the end user and public at large. To avoid misunderstanding there should be transparency about genetic ancestry inference assumptions and techniques. Claims must be supported by evidence that is clearly communicated. Effective translation of genetic ancestry inferences for the public requires acknowledgment of historical, social and cultural contexts and constructs of ancestry, race, and ethnicity. The public should be educated on the topic of genetic ancestry inference. Those engaging in genetic ancestry inference should promote education and inform the public.

This guideline is relevant to advertising, research publications, press releases, and other communication of genetic ancestry inference. Reasonable expectations of “end users” should include not only test-takers or roots-seekers and their immediate family members but also, for example, policymakers who might consider genetic ancestry inferences and the use of the results in litigation and administrative proceedings; the press and social media users whose understandings or misunderstandings have the potential for easy amplification and influence; members of the legal profession (such as attorneys and judges) who might proffer or encounter results in novel contexts; and educators. The guideline is important to minimize misrepresentations of genetic ancestry testing and inference and to protect consumers of genetic ancestry testing from bogus claims. Genetic ancestry information is often presented as exciting, revealing, and/or a challenge to existing knowledge, and this guideline is important by underscoring the need to not oversell or overstate results to the public. This guideline urges researchers, companies, and users to avoid embellishing claims through hypothetical storytelling used for dramatic effect, including such claims in press releases, news media, film, television, and other media.

The recommendations regarding communication and translation in this guideline stem from a fundamental difference of opinion regarding the impact of genetic ancestry inference/estimation. One area of agreement among the roundtables’ diverse stakeholders was the need for greater transparency and education, which hopefully will enable consumers and prospective study participants (whom researchers might offer opportunities to receive genetic ancestry information as a study benefit) to make informed decisions about whether to obtain an ancestry test. Communication and education are shared responsibilities that can be fulfilled in several ways but are dependent upon available resources. This guideline underscores the importance of ongoing efforts to improve communication and education while acknowledging the tensions commonly observed between those in marketing departments seeking to puff products and services with colorful commentary to increase sales and interest and the perceived “dull” or black-and-white scientific state of knowledge. Table 3 provides a comprehensive, but not necessarily exhaustive, list of various types of information that can be learned from genetic ancestry testing, which could inform the development or enhancement of educational tools for consumers and other users of genetic ancestry information.

**Table 3:** What can an individual learn from genetic ancestry testing?

Many things can be learned, with the strongest evidence coming from combining across genetic systems and with non-genetic evidence; however, DNA often captures information not recorded elsewhere		
a	info	DNA profile/genotype
	data	indels, short tandem repeats (STRs), SNPs from fragment analysis, gene chips (arrays), or sequencing (Sanger or next-generation)
b	info	Paternity/maternity tests
	data	autosomal STRs or genome-wide chips
	limits	availability of samples
	adv	parents and offspring share exactly half their autosomal genomes identical-by-descent, so inference is straightforward given sufficient information (marker variability or number)
c	info	Genomic kinship in any ancestral line (out to ∼fourth cousins)
	data	high-density autosomal genotypes/whole-genome sequence
	eg	identification of cryptic relatives^51^
	eg	complex pedigree reconstruction^52^
	eg	confirmation of illegitimate descents from Kaiser Wilhelm I of Germany 1797–1888, from autosomal genomic sharing (http://www.agnessecretprincess.com/)
	limits	cannot tell exact degree of cousinship; cannot detect many relationships beyond ∼ fifth cousins; some deeper relationships share considerably more DNA than average—high variance in sharing
	adv	recovers relationships in lineages other than the patriline and matriline
d	info	Genetic relationships in the male line within last ∼1,000 years (descent from common patrilineal ancestor)
	data	high-resolution Y chromosome genotypes/sequence
	eg	confirmation Sally Hemmings’s child was fathered by Thomas Jefferson^53^
	eg	shared descent of majority of Cohanim from patrilineal ancestor^54^
	eg	confirmation of Napoleon’s relics from patrilineal relative^55^
	eg	descent of ∼90% of males with surname Attenborough from a common male line ancestor^56^
	limits	no information about other ancestral lineages; confidence in closeness of relationship varies by information content of markers
	adv	records relationships over tens of generations not captured elsewhere in genome
e	info	Genetic relationships in the female line within last ∼1,000 years (descent from common matrilineal ancestor)
	data	high-resolution mtDNA genotypes/sequence
	eg	identity of skeleton of King Richard III, from match to distant matrilineal relatives^57^
	eg	identity of remains of Tsar Nicholas II of Russia and family in Ekaterinburg, from match to Prince Philip^58^^,^^59^^,^^60^
	eg	many examples (http://www.isogg.org/wiki/Success_stories)
	limits	no information about other ancestral lineages; relatively slow mutation rate means common ancestor can be >1,000 years ago
	adv	records relationships over tens of generations not captured elsewhere in genome; this lineage is often poorly detailed in paper records
f	info	Prehistoric ancestry of male line (inferred origins 1–100 kya)
	data	high-resolution Y chromosome genotypes/sequence
	eg	discovery of African male line ancestry for Yorkshire family^61^
	eg	evidence for Norse Viking male line ancestry in men across the British Isles carrying R1a-S443^62^^,^^63^^,^^64^^,^^65^
	limits	only one ancestral line; confidence in inference varies as information content of markers, size, and coverage of databases and actual phylogeographic patterns—some haplotypes are geographically very circumscribed, whereas others are more widespread
	adv	strong population genetic structure can allow fine-scale ancestry inference; retains information over many more generations than autosomal DNA
g	info	Prehistoric ancestry of female line (inferred origins 1–100 kya)
	data	high-resolution mtDNA genotypes/sequence
	eg	Native American matrilineal ancestry in majority of Colombian and other Mestizos in Latin America^66^
	eg	Princess Diana of Wales’s matrilineal Indian roots (https://www.thetimes.co.uk/article/revealed-the-indian-ancestry-of-william-ldvsjmc9w59)
	eg	matrilineal East Asian ancestry in a subset of Ashkenazi Jews^67^
	limits	only one ancestral line; confidence in inference varies as information content of markers, size and coverage of databases and actual phylogeographic patterns—weaker population genetic structure than Y means inference typically less fine-scale
	adv	retains information over many more generations than autosomal DNA; paper records very rare for deep matrilineal genealogy
h	info	Overall continental ancestry composition/components (%)
	data	high-density autosomal genotypes/whole-genome sequence
	eg	Native American genomic ancestry in Afro-Caribbean and Mestizo populations^68^^,^^69^^,^^70^
	eg	African ancestry in European Americans varies by state^71^
	eg	European ancestry in the Greenlanders^72^
	limits	large variation in amount of DNA inherited from different ancestors; beyond about seven generations genetic ancestors do not include all paper genealogy ancestors, only reflects proportion of pedigree ancestry further back; the confidence of particular percentage ancestry estimates is unclear
	adv	individual segments chronicle ancestry not recorded elsewhere in genome; DNA can reveal overall ancestry mix, irrespective of pedigree ancestors
i	info	Within continental ancestry composition/components (%)
	data	high-density autosomal genotypes/whole-genome sequence
	eg	inference of genetic differentiation between inhabitants of the English counties of Devon and Cornwall^73^ or Scottish cities of Aberdeen and Dundee^74^
	eg	inference of village of origin in European isolated populations^75^
	eg	Scandinavian, British, and East European ancestry among European Americans varies by state^71^
	limits	large variation in amount of DNA inherited from different ancestors; beyond about seven generations genetic ancestors do not include all paper genealogy ancestors, only reflects proportion of pedigree ancestry further back; the confidence of particular percentage ancestry estimates is unclear; more difficult for finer-scale inference, but see Gilbert et al.^74^ for potential future resolution
	adv	individual segments chronicle ancestry not recorded elsewhere in genome
j	info	Descent from specific historical ancestor
	data	high-resolution Y chromosome genotypes/sequence
	eg	identification of men patrilineally descended from Sir John Stewart of Bonkill c1245–1298, using marker S781, and Stewart YSNP haplotype; shared ancestry of many unpedigreed Stewarts from this lineage (http://www.s781.org/)
	eg	identification of men patrilineally descended from the lineage of John MacDonald of Islay, c1310–1386 (and thus likely Somerled) using marker YP326 and haplotype; shared ancestry of MacDonald clan chiefs and many unpedigreed MacDonalds from this lineage (https://clandonaldusa.org/index.php/dna-after-somerled)
	eg	identification of men descended from the lineage of Iain Cam MacGregor of Glenorchy, c1335–1390 using marker S690; shared ancestry of cadet branches of Clan Gregor and many unpedigreed MacGregors from this lineage (http://themacgregordnaproject.blogspot.com/2017/)
	limits	there are different degrees of certainty arising from particular genealogies available for discovery
	adv	very few people have 500+ year pedigrees, allows anyone to test for a connection to those who do; determines non-paternities
k	info	Parental consanguinity
	data	high-density autosomal genotypes/whole-genome sequence
	eg	inference of recent ancestral inbreeding^76^
	eg	inference of demographic history of ancestors^77^
	eg	inference of incest^78^
	limits	cannot infer exact degree of parental kinship
	adv	documents parental relationships in any ancestral line
Depending on the test taken, the genetic raw data may also provide non-ancestry related information, if subjected to an analysis to extract such information		
l	info	Recreational genomic information
	data	high-density autosomal genotypes/whole-genome sequence
	eg	carrier status for recessive traits, e.g., red hair
	eg	genetic risk of complex trait, e.g., baldness, asparagus pee
	limits	complex trait prediction is as yet of limited accuracy for most individuals
	adv	in some cases, individuals will be able to estimate chances of offspring carrying traits
m	info	Health information
	data	high-density autosomal genotypes/whole-genome sequence
	eg	carrier status for recessive disease, e.g., cystic fibrosis
	eg	genetic risk of complex disease, e.g., breast cancer
	eg	genetic risk of adverse drug reaction
	limits	complex disease prediction is as yet of limited accuracy for most individuals
	adv	accurate prediction will empower precision medicine

Further explanation is needed for this guideline to emphasize that this is a rational recommendation based on disagreement among stakeholders about specific details. This guideline is vulnerable to subjective interpretation of what constitutes transparent communication and education. Subjective interpretation allows for flexibility necessary to garner broad support for the guideline and multiple ways with which to fulfill the responsibilities set by the guideline. However, a third party and/or non-partisan or bipartisan review of content and form of communications over time could alleviate the limitation.

This guideline lends itself to four potential next steps. Firstly, the next step toward establishing a set of best practices would be to define standards (e.g., what constitutes accuracy, transparency, acknowledgment of social factors, and education). Secondly, it would be prudent to take steps to avoid hype when reporting research findings and to take corrective actions when research findings or inferences reported by others are inflated or otherwise misinterpreted. Thirdly, it is appropriate for those engaged in genetic ancestry inference to treat every transaction and study participation as a “teachable moment.” Additionally, a roundtable discussion with communication scholars and science journalists might be useful to address the aforementioned limitation of the guideline.

## Discussion

By way of a process of consensus building through two roundtables involving dozens of stakeholders, we have developed a set of broadly agreed upon guidelines that form the basis for more specific recommendations about the use of genetic ancestry inference. Development and operationalization of such recommendations will require substantially more discussion by stakeholders and beyond. These two roundtables stemmed from an earlier working group and identify a wide range of implications involving heterogeneous stakeholders. It is only through continued engagement that the details (of who else, how, and in what contexts these guidelines will be relevant) can even be established. At present, we hope that those conducting genetic ancestry inference, be they researchers or DTC testing companies, consider adopting and continuing to contribute to the refinement and implementation of these guidelines. Most important will be their leadership in operationalizing these guidelines with the support of scholars and policymakers. As with all guidelines, we expect them—like genetic ancestry inference itself—to change over time via stakeholder-engaged processes. Most specifically, several major governmental bodies and regulators have important roles to play in supporting and promulgating a number of key practices and shared resources.

As an attempt at full transparency, we have attested to the imprecision of both our processes to develop these guidelines and the guidelines themselves. Because genetic ancestry inference tests and practices are moving targets, we must balance precision of our guidelines with the need for those guidelines to be both responsive and durable. Again, only through empirical study and expanding to new issues from new stakeholders will we have a chance at maintaining or increasing any sense of precision in these and future guidelines.

We have highlighted several limits to our processes and have acknowledged both flaws in the approach taken and caveats with the resulting deliverables. For example, the structure of Roundtable I (small group discussions with diverse stakeholder representation in each small group followed by full discussion by all participants) provoked noticeable frustrations among participants, as compromises reached in small group discussions were reopened and issues with particular statements were rehashed and renegotiated among the full group discussions. This inefficiency likely influenced the tally of votes on “consensus” statements. Also illustrative is our admission of ways in which the guidelines from Roundtable II require further explanation and the details to be operationalized. Yet the fact that we anchor these guidelines with their limitations and dissents provides the important opportunity to both enhance future processes and most transparently continue to modify and hopefully improve these guidelines. For example, the evolution from Roundtable I to Roundtable II involved moving from a long list of fragmented statements with varying degrees of support to a smaller set of thematically coherent guidelines representing a process of consensus.

Since the roundtable discussions, scholarly work examining genetic ancestry inference, descriptions of human variation, and impacts of genetic ancestry testing have continued;^79^^,^^80^^,^^81^^,^^82^^,^^83^^,^^84^^,^^85^^,^^86^^,^^87^^,^^88^^,^^89^^,^^90^^,^^91^^,^^92^^,^^93^^,^^94^ industry practices for DTC genetic ancestry inference have changed (for a list of active and inactive DTC companies offering genetic ancestry testing, see https://isogg.org/wiki/List_of_DNA_testing_companies); and some genetic genealogy standards have emerged.^95^ Policy and scholarly developments—for example, regarding privacy harms,^96^ data justice and dataveillance concerns,^97^ attention brought to Indigenous data sovereignty,^44^ and calls in the United States for comprehensive (rather than sector-specific) approaches to personal data protection^98^ as well as modernized rules for data access and increased interoperability^99^^,^^100^^,^^101^^,^^102^—have substantial implications for the refinement and implementation of guidelines for genetic ancestry inference and addressing matters of genetic privacy. In addition to the changing state laws for genetic information privacy specifically (e.g., Florida’s Protecting DNA Privacy Act, H.B. 1189, enacted in 2021; Utah’s Genetic Information Privacy Act, S.B. 227, enacted in 2021; Wyoming’s Genetic Data Privacy Act, H.B. 86, enacted in 2022; and Kentucky’s Genetic Information Privacy Act, H.B. 502, enacted in 2022) and personal data protection generally (e.g., the California Privacy Rights Act, Proposition 24, approved by CA voters in 2020; the Colorado Privacy Act, S.B. 21-1980, enacted in 2021; and the Virginia Consumer Data Protection Act, H.B. 2307/S.B. 1392, enacted in 2021), agencies such as the Federal Trade Commission have been increasingly active in ensuring that data practices are not unfair or deceptive or unreasonably lax with regard to privacy and security measures. Those engaged in genetic ancestry inference—whether as members of the DTC industry or academic research—will need to pay careful attention to shifting data governance expectations of individuals and obligations imposed implicitly or explicitly by policymakers.

While this report reflects efforts undertaken several years ago, the issues remain salient. Moreover, the deliverables (i.e., the “consensus” statements and preliminary guidelines) from the impassioned roundtable discussions represent an ongoing need to address current scientific and societal challenges with genetic ancestry inference. These guidelines were never intended to serve as an endpoint but, rather, as a crucial step toward the development, implementation, and continued improvement of best practices. It is our hope that this report will assist in ongoing scholarly, industry, and policy discussions for the advancement of science and broad human benefit.

## Diverse stakeholders in the roundtable discussions

In addition to the authors in the writing group, the participants in the roundtable discussions included Thomas W. Athey, Mathew Barber, Jessica Bardill, Terry Barton, Blaine Bettinger, Deborah Bolnick, Vence Bonham, Katherine Borges, Joann Boughman, Esteban Burchard, Carlos Bustamante, Jake Byrnes, Shannon Stewart Christmas, Mike Dougherty, Carla Easter, Alice M. Fairhurst, Peter Förster, Elise Friedman, Malia Fullerton, Jonathan Gitlin, Bennett Greenspan, Tim Janzen, Kathryn Johnston, Rick Kittles, Sandra Soo-Jin Lee, Sally Lehrman, Jeffrey C. Long, Rita Anne Lopez, Joe McInerney, Yolanda T. Moses, Joanna Mountain, Alondra Nelson, Jean-Chretien Norreel, John Novembre, Pilar N. Ossorio, Harry Ostrer, David Pike, Terry Powell, Jenny Reardon, Casey Ross-Pethernick, Laura Lyman Rodriguez, Charles Rotimi, Ali M. Salih, Pamela Sankar, Theodore Schurr, Alexandra Shields, Mark Shriver, Eugene J. Silva, Lindsay Smith, Megan Smolenyak, Bryan Sykes, Kim Tallbear, Noah Tamarkin, Ann Turner, Marilyn Vann, James Larry Vick, Spencer Wells, Ron Whitener, Jun J. Yang, and Laurie Zoloth.

## Data and code availability

This effort did not generate or analyze datasets or code except for the tally of votes during the roundtables, which are included in the tables of this publication.
